# Siglec-15-induced autophagy promotes invasion and metastasis of human osteosarcoma cells by activating the epithelial–mesenchymal transition and Beclin-1/ATG14 pathway

**DOI:** 10.1186/s13578-022-00846-y

**Published:** 2022-07-16

**Authors:** Bingxin Zheng, Keliang Song, Lingling Sun, Yang Gao, Yan Qu, Chongmin Ren, Peng Yan, Wenfang Chen, Wei Guo, Chuanli Zhou, Bin Yue

**Affiliations:** 1 Department of Orthopedic Oncology, The Affiliated Hospital of Qingdao University, No. 59 Haier Road, Qingdao, 266000 People’s Republic of China; 2 Department of Pathology, The Affiliated Hospital of Qingdao University, Qingdao, People’s Republic of China; 3 Medical Department, The Affiliated Hospital of Qingdao University, Qingdao, People’s Republic of China; 4 Industrial Investment Department, Haier, Qingdao People’s Republic of China; 5 Department of Physiology, Medical College of Qingdao University, Qingdao, People’s Republic of China; 6 Musculoskeletal Tumor Center, Peking University People’s Hospital, Beijing, People’s Republic of China; 7 Department of Spinal Surgery, The Affiliated Hospital of Qingdao University, No. 59 Haier Road, Qingdao, 266000 People’s Republic of China

**Keywords:** Sialic acid-bound immunoglobulin lectin 15 (Siglec-15), Autophagy, Metastasis, Osteosarcoma, Beclin-1

## Abstract

**Background:**

Pulmonary metastasis is the main cause of poor prognosis in osteosarcoma. Sialic acid-bound immunoglobulin lectin 15 (Siglec-15) has been demonstrated to be obviously correlated with pulmonary metastasis in osteosarcoma patients. However, the effect of Siglec-15 on autophagy in osteosarcoma remains unclear, while the role and mechanism of Siglec-15-related autophagy in lung metastasis also remain unknown.

**Methods:**

The expression levels of Siglec-15 and Beclin-1 were detected in osteosarcoma tissues using immunohistochemistry (IHC). The effect of Siglec-15 on metastasis was investigated using Transwell, wound healing and animal experiments with osteosarcoma cells. Corresponding proteins were confirmed using Western blotting when Siglec-15 or Beclin-1 was silenced or overexpressed. Changes in autophagy and the cytoskeleton were detected using immunofluorescence and transmission electron microscopy.

**Results:**

Siglec-15 and Beclin-1 expression was evaluated both in lung metastases and in patients who presented with pulmonary metastasis of osteosarcoma. Immunoprecipitation experiments revealed that Siglec-15 interacts directly with Beclin-1, an important autophagic protein. Moreover, loss of Siglec-15 distinctly inhibited autophagy and reduced Beclin-1/ATG14 expression. The decreased invasion and migration caused by Siglec-15 silencing could be reversed by Beclin-1 overexpression. Additionally, autophagy can promote the epithelial–mesenchymal transition (EMT) and affect cytoskeletal rearrangement, which was confirmed by overexpression or silencing of Beclin-1.

**Conclusions:**

These findings confirmed the role of Siglec-15 in the regulation of autophagy and elaborated the relationship and mechanisms between autophagy and the metastasis of osteosarcoma cells.

**Supplementary Information:**

The online version contains supplementary material available at 10.1186/s13578-022-00846-y.

## Background

As the most common malignant bone tumor, osteosarcoma occurs mainly in children and adolescents [1], and the pulmonary metastasis rate of osteosarcoma patients is approximately 20–30% [2]. Patients with lung metastases tend to be resistant to chemotherapy and have a poor 5-year survival of only approximately 10–20% [3]. Therefore, targeted treatment for pulmonary metastasis is important to improve the prognosis of patients with osteosarcoma. With the development of immunotherapy, many immunosuppressive molecules, such as programmed cell death ligand 1 (PD-L1), programmed cell death ligand 2 (PD-L2), programmed cell death 1 (PD-1), T-cell immunoglobulin and mucin domain-3 (TIM-3) and lymphocyte-activation gene 3 (LAG-3), have been studied in depth [4–6]. However, these studies focus mainly on their expression and functions in immune cells, and there are few studies on their effects on the intrinsic function and mechanism of tumor cells. Sialic acid-bound immunoglobulin lectin 15 (Siglec-15) was previously found to play a role in osteogenic differentiation and microbial infection [7], but was later found to have an immunosuppressive function [8]. Siglec-15 was reported to be highly expressed in various solid tumors [9, 10], while its expression was limited in normal tissue. Siglec-15 is partially homologous with PD-L1 on the structure, while the relationship between Siglec-15 expression and PD-L1 expression in some cancers is mutually exclusive [8]. Siglec-15 may function similarly to PD-L1, both in the immune microenvironment and in tumor cells [11]. Hence, Siglec-15 may play important roles and functions in some tumors, especially in tumor patients with low expression of PD-L1. For osteosarcoma, a recent study suggested that Siglec-15 is involved in pulmonary metastasis [12]. To the best of our knowledge, there are no studies reporting on the effect of Siglec-15 on autophagy in osteosarcoma, nor have studies on the role of autophagy in metastasis been performed.

In this study, both Siglec-15 and Beclin-1 expression were detected in primary and metastatic osteosarcoma lesions. The roles of Siglec-15 in the migration, invasion and autophagy of osteosarcoma cells were researched both in vitro and in vivo. In addition, we explored the role and potential mechanism of Siglec-15-related autophagy in the process of pulmonary metastasis in osteosarcoma patients.

## Materials and methods

### Tissue samples and patient information

Formalin-fixed and paraffin-embedded primary tissue specimens of histopathologically diagnosed osteosarcoma and matched pulmonary metastasis lesions were obtained from the Department of Pathology of the Affiliated Hospital of Qingdao University and the Musculoskeletal Tumor Center of Peking University People’s Hospital. Informed consent was obtained from all patients. The study was also approved by the ethics committees of the Affiliated Hospital of Qingdao University and Peking University People’s Hospital.

### Cell culture and antibodies

The osteosarcoma cell lines U2OS and KHOS were acquired from the American Type Culture Collection (ATCC, MD, USA). U2OS and KHOS cells were maintained in Roswell Park Memorial Institute (RPMI)-1640 supplemented with 10% fetal bovine serum and 1% antibiotics, and the used cell lines were cultured at 37 °C with 5% CO_2_.

Antibodies used in the experiments were as follows: antirabbit Siglec-15 antibody (AP11503b) (Abcepta, Suzhou, China ), antirabbit Beclin-1 antibody (ab210498) (Abcam, Cambridge, UK), antirabbit P62 antibody (ab207205) (Abcam, Cambridge, UK), antirabbit ATG14 antibody (bs-7462R) (Bioss, Beijing, China), antirabbit LC3A/B antibody (12,741) (CST, MA, USA), antirabbit LC3B antibody (3868) (CST, MA, USA), antirabbit E-cadherin antibody (bs-1519R) (Bioss, Beijing, China), antirabbit N-cadherin antibody (bs-1172R) (Bioss, Beijing, China), antirabbit Vimentin antibody (bs-0756R) (Bioss, Beijing, China), antirabbit MMP-9 antibody (13,667) (CST, MA, USA), antirabbit GAPDH antibody (AB0037) (ShareBio, Shanghai, China), antirabbit IgG (Hazel) labeled antibody (AB0101) (ShareBio, Shanghai, China), the RhoA activation assay (ab211164) (Abcam, Cambridge, UK).

### Gene knockdown and ectopic expression

ShSiglec-15 lentiviruses were acquired from RiboBio (Guangzhou, China). The Siglec-15 shRNA sequences were as follows: #1, sense strand 5′-CTACGGAGAACTTGCTCAA-3′ and antisense strand 5′-TTGAGCAAGTTCTCCGTAG-3′; #2, sense strand 5′-GGCCCAGGAGTCCAATTAT-3′ and antisense strand 5′-ATAATTGGACTCCTGGGCC-3′. ShSiglec-15 stably expressing cells were acquired using 2 mg/ml puromycin selection.

SiBeclin-1 was purchased from RiboBio (Guangzhou, China). The sequences targeting Beclin-1 were as follows: #1, 5′-CAGGAUGAUGUCCACAGAATT-3′ (sense) and 5′- UUCUGUGGACAUCAUCCUGGC-3′ (antisense); #2, 5′-CAAGUUCAUGCUGACGAAUTT-3′ (sense) and 5′-AUUCGUCAGCAUGAACUUGAG-3′ (antisense); #3, 5′- CGUGGAAUGGAAUGAGAUUTT-3′ and (sense) and 5′-AAUCUCAUUCCAUUCCACGGG-3′ (antisense). SiRNAs were transfected into osteosarcoma cells using Lipofectamine 3000 (Invitrogen, CA, USA).

The plasmid containing Beclin-1 and Siglec-15 cDNA or negative control were obtained from RiboBio (Guangzhou, China) and used to transfect KHOS cells with Lipofectamine 3000 (Invitrogen, CA, USA). The medium was replaced after 24 h of incubation. The corresponding functional assays were carried out after 24 h, and protein was extracted after 48 h of transfection.

### Transwell assays

Cells were planted into chambers coated with or without Matrigel (Corning, NY, USA, 354480, 3422) for migration capacity and invasion ability analysis. After culturing for 24 h, cells that passed through Transwell chambers were fixed with methanol and stained with Giemsa staining solution. The number of migrated cells was counted under a microscope in five random fields per well.

### Wound healing assays

U2OS and KHOS cells were plated into 6-well plates for cell mobility evaluation. When the cells were cultured to 90% confluence, a scratch was made carefully across the plates by using a 200-µl sterile pipette tip. Images were captured after incubation for 0 and 24 h and analyzed using ImageJ.

### RNA-sequencing and bioinformatics analyses

Total RNA of each sample was extracted according to the instruction manual of the TRIzol Reagent (Life Technologies, CA, USA). Subsequently, RNA qualification, library construction and sequencing were conducted by Beijing Biomarker Technologies Co., Ltd. (Beijing, China). The abundant differences in gene expression between these samples were determined based on the ratio of the FPKM values (fragments per kilobase of exon per million fragments mapped) by Cufflinks software [13]. The false discovery rate (FDR) control method was used to recognize the threshold of the *P* value in multiple tests to calculate the significance of the differences. Only the genes with an absolute value of log_2_ ratio ≥ 2 and FDR significance score < 0.01 were used for further analysis.

The potential interaction between Siglec-15 and Beclin-1 was investigated by bioinformatic analysis. Broadly, we typed the target gene names via the website (http://genemania.org/) and performed the corresponding settings to investigate the associations between these genes.

### Western blotting and GTPase assay

Briefly, cell lysates were acquired from the corresponding groups using radioimmunoprecipitation assay (RIPA) lysis buffer. The proteins were separated on 7.5–15% sodium dodecyl sulfate-polyacrylamide gel electrophoresis (SDS-PAGE) gels using a NuPAGE system and transferred to polyvinylidene difluoride membranes. Then, the membranes were incubated with the corresponding primary antibodies overnight at 4 °C after blocking for 1 h. The protein bands were visualized by electrochemiluminescence. The GTPase assay was implemented according to the kit instructions.

### Immunoprecipitation

A suitable amount of antibody was added to the cell lysis solution and then incubated for 3 h at 4 °C. Subsequently, protein A-agarose (Vigorous Biotechnology, Beijing, China; P007) was incubated with the solution for 1 h. The immune precipitates were washed three times using a lysis solution followed by elution with SDS loading buffer. The eluent was subjected to Western blotting.

### In vitro beclin-1-Siglec-15 pulldown

GST protein interaction pull-down kits were obtained from Thermo Fisher (MA, USA). Bind recombinant Beclin-1 protein to glutathione high-capacity magnetic agarose beads according to the manufacturer’s instructions. KHOS osteosarcoma cells at 80% density were lysed in the above co-immunoprecipitation buffer for 15 min at 4 °C and centrifuged at 15,000×*g* for 15 min. While the cells were lysed, an appropriate amount of the bead slurry was blocked with 5% BSA in lysis buffer for 10 min at 4 °C. The lysed protein was then incubated with the blocked bead slurry for 60 min at 37 °C. After protein binding, resuspend in 1× SDS sample buffer and boil for 5 min, separate by SDS-PAGE, and probe by western blot.

### Transmission electron microscopy(TEM)

For the TEM assay, the cells of the corresponding groups were digested with 0.25% trypsin. Then, 1.5% glutaraldehyde was used to fix the cells at 4 °C for 6 h. Ultrathin Sect. (100 nm) were stained with uranyl acetate and lead citrate and then examined under a TEM (H-600; Hitachi, Tokyo, Japan).

### Immunohistochemistry assay

Immunohistochemistry (IHC) staining was conducted as described previously [14]. Briefly, the paraffin sections were deparaffinized and exposed to the corresponding primary antibodies overnight at 4 °C. Then, the sections were reacted with secondary antibody for 30 min at 37 °C. The positive staining score was defined as the sum of the staining percentage (0: 0% positive; 1: < 5% positive; 2: 5–50% positive; and 3: > 50% positive) and staining intensity (0: none; 1: weak; 2: moderate; and 3: intense). More than 10 representative fields (400× magnification) were used for the assay. The immunostaining was evaluated by two independent pathologists who were unfamiliar with the clinical specimens.

### Immunofluorescence assay

Cells were plated onto coverslips in 6-well plates with corresponding treatments. Next, 4% paraformaldehyde was used to fix cells for 20 min. Then, the cells were incubated with 0.1% Triton X-100 for 5 min. For the immunofluorescence assay of the cytoskeleton, the coverslips shielded from light were cultivated with phalloidin-iFluor 594 reagent (ab176757) (Abcam, Cambridge, UK) for 45 min at room temperature. For the immunofluorescence assay of LC3, the cells were incubated with antiLC3 antibody overnight at 4 °C and then washed 3 times with phosphate buffered saline (PBS). The cells were exposed to a suitable secondary antibody at room temperature. 4′,6-Diamidino-2-phenylindole (DAPI) staining was used for nuclear staining in the immunofluorescence assay. All the cells were ultimately observed using confocal microscopy (FV10i, Olympus, Tokyo, Japan).

### Generation of xenografts

For the analysis of the effect of Siglec-15 knockdown on the metastatic capacity of KHOS cells, 3 × 10^6^ cells (KHOS-shSiglec-15 or KHOS-shNC) were intravenously injected into the tail vein of 6-week-old female BALB/c nude mice (Vitalriver, Beijing, China). All mice were sacrificed after 30 days. The lungs of mice were routinely obtained, fixed and prepared for subsequent hematoxylin-eosin (H&E) and immunohistochemical staining. Then, the number of pulmonary metastatic nodules was quantified. All the animal care and processes involved in this experiment were enforced in conformity with the National Institutes of Health Guide for the Care and Use of Laboratory Animals.

### Statistical analysis

Data are presented as the mean ± standard deviation (SD). SPSS v.21.0 software (SPSS, Chicago, IL, USA) was used for statistical analyses. *χ*^2^ test and Student’s *t*-test were used for statistical evaluation. A *P* value < 0.05 was considered to indicate significant differences.

## Results

### Siglec-15 and Beclin-1 expression is closely related to lung metastases of osteosarcoma

**Fig. 1:**
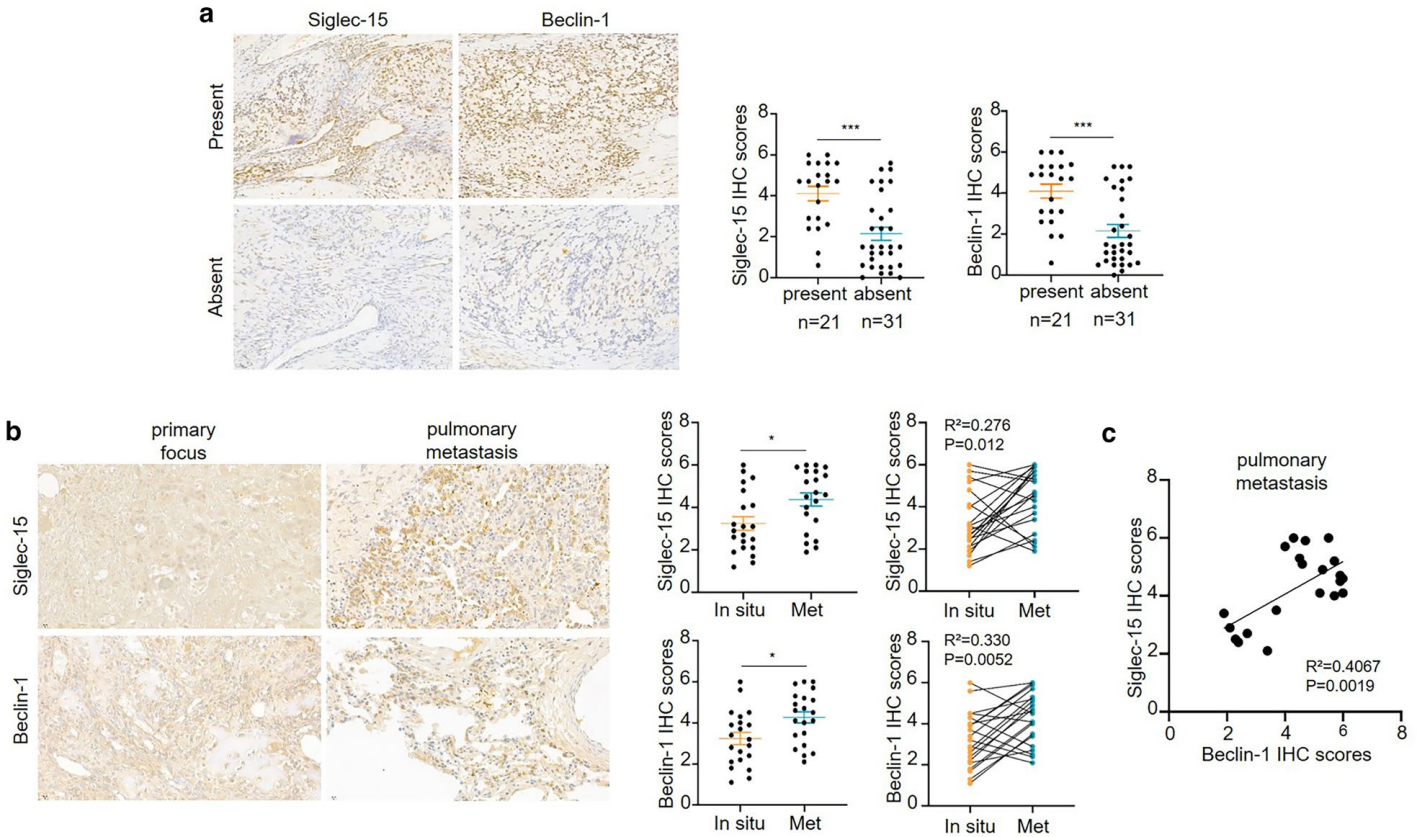
Siglec-15 and Beclin-1 expression is closely related to lung metastases of osteosarcoma. **a** Contrasting with those without lung metastases, osteosarcoma patients with lung metastasis had higher expression of Siglec-15 and Beclin-1 in the primary tumor using immunohistochemistry. **b** The immunohistochemistry results indicated that significantly higher expression levels of Siglec-15 and Beclin-1 were detected in lung metastases. **c** Siglec-15 and Beclin-1 expression levels show a positive correlation in lung metastases. Representative immunohistochemical images are shown at ×200 and ×400 magnification. Data are presented as the mean ± SD. *P < 0.05

IHC experiments were conducted to investigate Siglec-15 and Beclin-1 expression in osteosarcoma. First, the Siglec-15 and Beclin-1 proteins were detected in primary osteosarcoma specimens (n = 52), and the primary osteosarcoma specimens were divided into two groups according to the presence or absence of pulmonary metastasis at the time of surgical resection (evidenced by medical imaging). Both Siglec-15 and Beclin-1 were highly expressed in the osteosarcoma group presenting with lung metastasis compared to the group without lung metastasis (Fig. 1a). However, the expression of Siglec-15 and Beclin-1 had no correlation with sex, age, tumor location or histological classification (Table 1). Second, we further examined the expression of Siglec-15 and Beclin-1 in paired primary osteosarcoma samples and corresponding pulmonary metastases (n = 21 pairs). The expression of Siglec-15 in the lung metastases was significantly higher than the expression of Siglec-15 in the primary lesions, while Beclin-1 was also expressed more strongly in the lung metastases (Fig. 1b). Moreover, further analysis revealed a positive correlation between the expression of Siglec-15 and Beclin-1 in lung metastases (Fig. 1c). In conclusion, these results suggested that the expression of Siglec-15 and Beclin-1 was associated with pulmonary metastasis in osteosarcoma patients.

**Table 1:** Relationship between clinicopathologic parameters and Siglec-15 and Beclin-1 expression in human osteosarcoma tissues

Variables	N (52)	Siglec-15 expression			Beclin-1 expression		
		+	−	P	+	−	P
Age							
≤ 25	17	7	10	0.257	11	6	0.622
> 25	35	9	26		25	10	
Sex							
Male	33	11	22	0.598	23	10	0.924
Female	19	5	14		13	6	
Size (cm)							
≤ 5	15	5	10	1.000	13	2	0.161
> 5	37	11	26		23	14	
Histological classification							
Osteoblastic	20	5	15	0.610	17	3	0.149
Chondroblastic	20	6	14		12	8	
Others	12	5	7		7	5	
Main tumor location							
Limb bone	38	13	25	0.795	27	11	0.892
Spine	6	1	5		4	2	
Pelvis	8	2	6		5	3	
Lung metastasis							
Yes	21	11	10	0.005	18	3	0.034
No	31	5	26		18	13	

### Potential mechanisms that underlie the Siglec-15 associations with metastasis

**Fig. 2:**
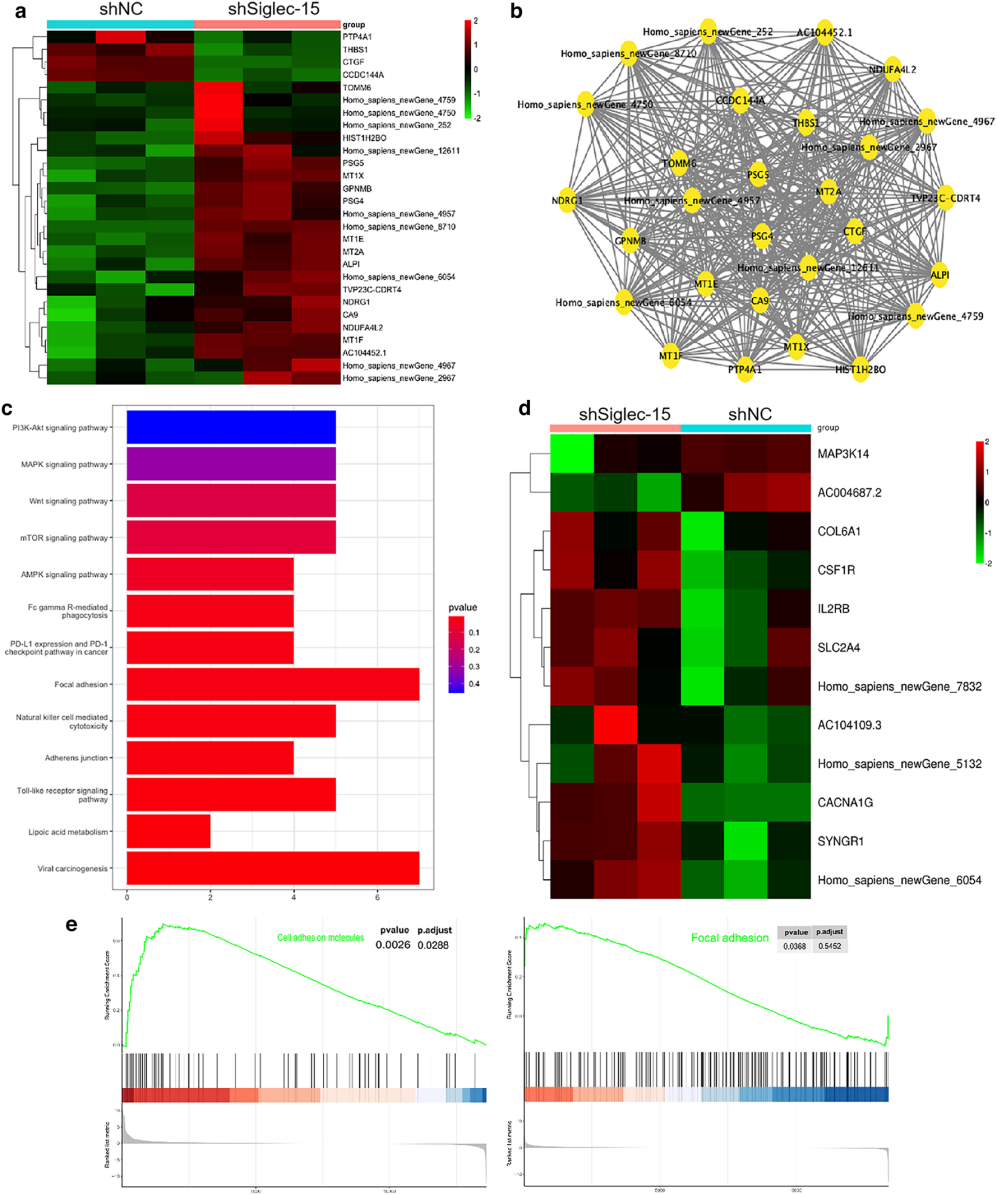
Potential mechanisms that underlie the Siglec-15 associations with metastasis. **a** Heatmap of representative differentially expressed gene resulting from the control group and Siglec-15 knockdown group through transcriptomic sequencing. **b** A gene map of coexpression of these differentially expressed genes. **c** KEGG pathway analysis of differentially expressed genes revealed Siglec-15 associations with cell autophagy and migration. **d** Heatmap of differentially expressed genes, based on autophagy-related pathway analysis, revealed Siglec-15-mediated autophagy in osteosarcoma cells. **e** GSEA of the genes associated with the focal adhesion and cell adhesion molecules in control group and Siglec-15 knockdown group

Transcriptomic analysis was used to examine the effect of Siglec-15 on osteosarcoma metastasis. After Siglec-15 knockdown in KHOS cells, the heat map of the top differentially expressed genes (versus the control group) was shown in Fig. 2a. A gene map of co-expression of these differentially expressed genes was also shown in Fig. 2b. The Kyoto Encyclopedia of Genes and Genomes (KEGG) pathway analysis revealed that the genes linked to focal adhesion, and adherens junction pathways were significantly involved in the Siglec-15 associations with metastasis (Fig. 2c). It is known that autophagy is a complex process of cell self-degradation, in which many signaling pathways are involved, including Wnt, mTOR, PI3K-Akt, MAPK [15–18], etc. Through KEGG analysis, we also found that Wnt, mTOR, PI3K-Akt, MAPK and AMPK signaling pathways were involved in the relationship between Siglec-15 and autophagy (Fig. 2c). Furthermore, based on these autophagy related pathways, heatmap analysis of differentially expressed genes revealed the potential genes that may be involved in Siglec-15-mediated autophagy (Fig. 2d). Additionally, the GSEA results also indicated that Siglec-15 was positively associated with focal adhesion and cell adhesion molecules (Fig. 2e). In brief, these transcriptomic-level findings supported that Siglec-15 depletion could inhibit the invasive tendency of osteosarcoma, and autophagy may be one of the important influencing factors.

### Siglec-15 depletion suppresses the migration and invasion of osteosarcoma cells in vitro

**Fig. 3:**
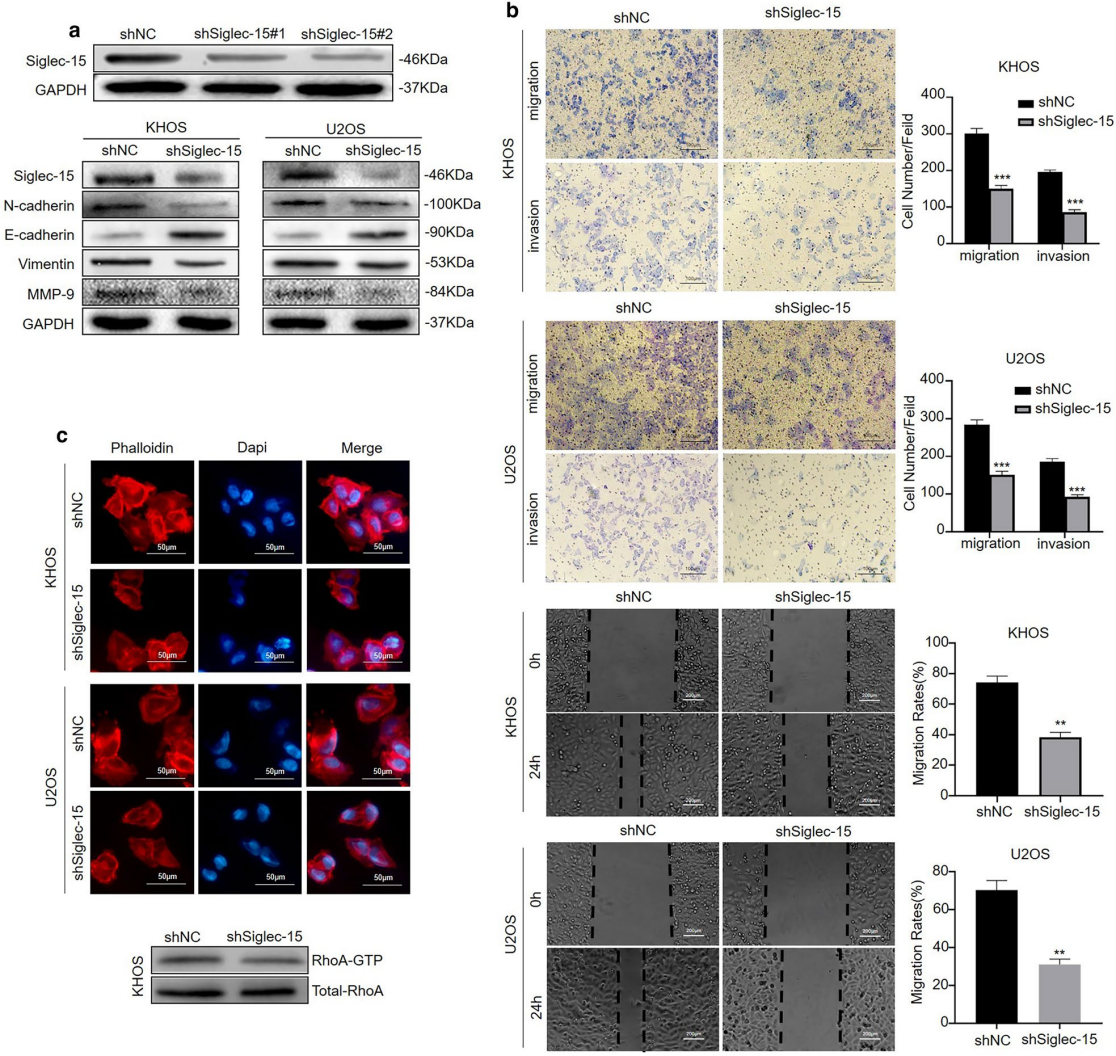
Siglec-15 knockdown suppresses the migration and invasion of osteosarcoma cells in vitro. **a** ShRNA sequences targeting Siglec-15 were used, and the knockdown efficiency was measured by Western blotting. Siglec-15 depletion inhibits epithelial–mesenchymal transition, as shown by Western blotting. **b** Transwell assays and wound healing assays indicate that Siglec-15 depletion significantly decreased the migration and invasive capacity of KHOS and U2OS cells. **c** Cytoskeletal assays show that more conspicuous lamellipodial protrusions were formed in the control cells than in the Siglec-15-depletion group. Representative images are shown. Cell nuclei are stained with DAPI. The scale bar represents 50 μm. Decreased RhoA activation was also observed in the Siglec-15-depletion group. All experiments were repeated three times. Data are presented as the mean ± SD. **P < 0.01, ***P < 0.001

ShRNA lentiviruses targeting Siglec-15 were used for the functional examination of Siglec-15 in osteosarcoma cells, and Siglec-15 expression was significantly inhibited in the Siglec-15 knockdown group (Fig. 3a). To ascertain the role of Siglec-15 in the migration and invasiveness of osteosarcoma cells, both Transwell assays and wound healing assays were conducted. The results indicated that Siglec-15 depletion significantly decreased the migration and invasive capacity of KHOS and U2OS cells (Fig. 3b). Because epithelial–mesenchymal transition (EMT) is also a crucial point involved in tumor metastasis [19], we examined the effect of Siglec-15 knockdown on EMT by Western blotting. E-cadherin, an epithelial marker, was increased in the shSiglec-15 group compared to the control group (Fig. 3a). Decreased expression of N-cadherin and vimentin, which are mesenchymal characteristics, was detected in the shSiglec-15 group (Fig. 3a). Matrix metalloproteinase-9 (MMP-9) was also decreased in the Siglec-15-depletion group (Fig. 3a). Conversely, the Siglec-15 expression recovery increased Vimentin and decreased E-cadherin levels, and induced EMT in shSiglec-15-KHOS cells (Additional file 2: Fig. S2a). The Siglec-15 expression recovery increased the migration and invasion of shSiglec-15-KHOS cells (Additional file 2: Fig. S2c). The quantification of Western blot results was shown in Additional file 1: Fig. S1a.

The cytoskeleton plays a vital role in cell migration [20]. As mentioned before, cytoskeletal rearrangement was involved in the potential mechanisms that underlie the Siglec-15 associations with metastasis. Cytoskeletal assay of KHOS and U2OS cells was visualized using confocal microscopy. As shown in the results, conspicuous lamellipodial protrusions were formed in the submembranous area of the control cells, but the opposite phenomenon was observed in the Siglec-15-depletion group, which represented the rearranged cytoskeleton and well-distributed F-actin in both KHOS and U2OS cells (Fig. 3c). Additionally, LIMK and cofilin, the downstream molecules of RhoA, are crucial regulators in the regulation of the actin cytoskeleton [20]. Since RhoA could regulate the phosphorylation of LIMK and cofilin, we detected changes in RhoA after Siglec-15 deletion. Decreased RhoA activation was observed in the Siglec-15-depletion group compared to the control group using the GTPase assay (Fig. 3c). These outcomes suggested that Siglec-15 expression could distinctly influence cytoskeletal rearrangement in osteosarcoma cells.

### Siglec-15 is involved in the regulation of autophagy

**Fig. 4:**
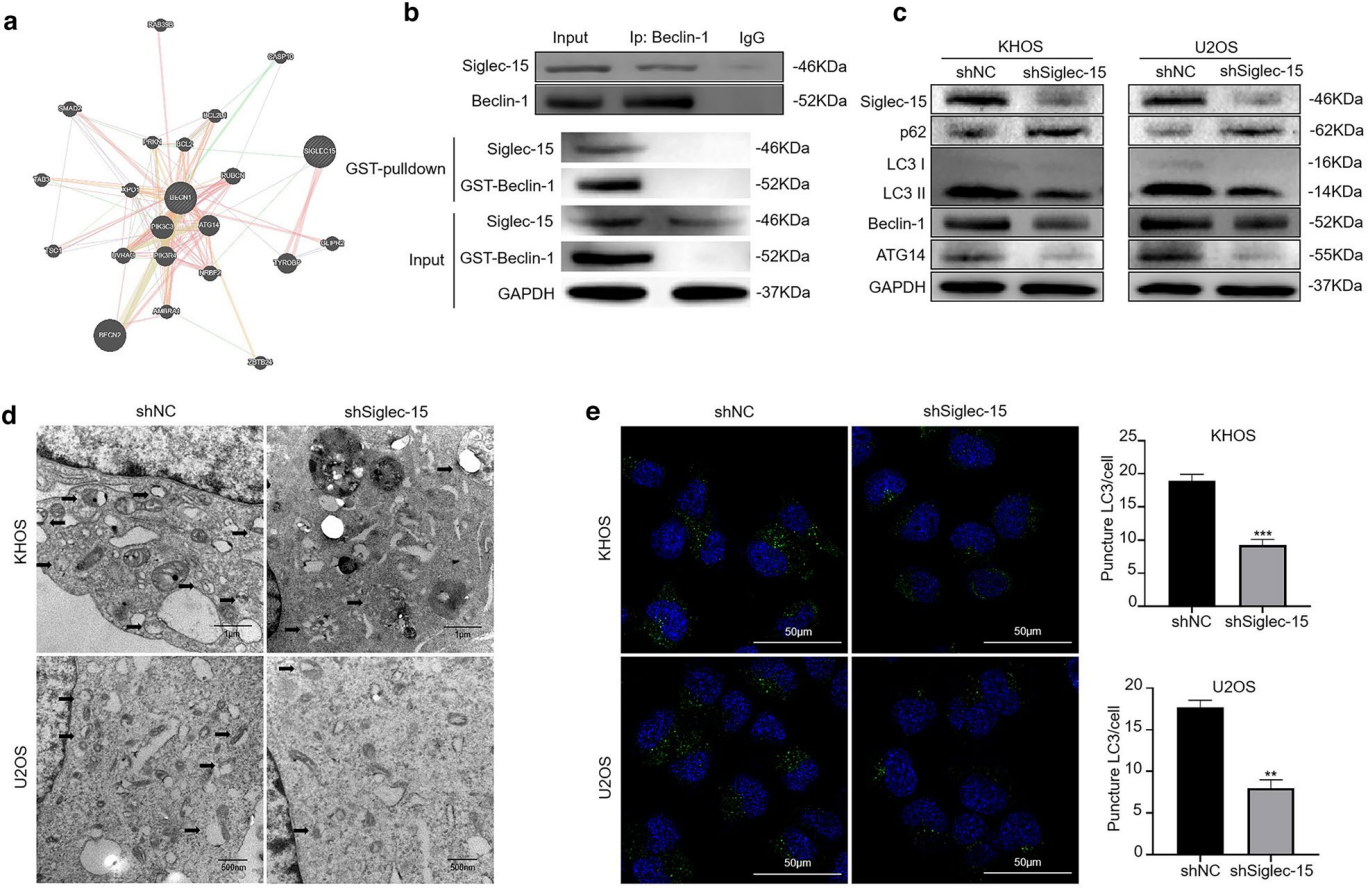
Siglec-15 inhibition diminishes the autophagy process by inactivating the Beclin-1/ATG14 pathway. **a** Bioinformatics prediction of the interaction between Siglec-15 and Beclin-1 (http://genemania.org/). **b** Immunoprecipitation was used to evaluate the interaction between Siglec-15 and Beclin-1 (up). Recombinant GST-Beclin-1 conjugated beads were used to examine relative amounts of co-precipitating Siglec-15 (down). **c** Western blotting was used to evaluate the expression of autophagy-related proteins, including Beclin-1, ATG14, LC3 and p62. **d** Representative TEM images present the ultrastructures during autophagy after Siglec-15 depletion. The images show more autophagic vacuoles (arrows) observed in control cells. **e** Punctate LC3 levels in the control and Siglec-15 inhibition groups are presented and quantified. Data are presented as the mean ± SD. of three independent experiments. **P < 0.01, ***P < 0.001

The bioinformatics prediction suggested that there may be an interaction between Siglec-15 and Beclin-1 (Fig. 4a). Subsequently, a coimmunoprecipitation study was used to test this prediction. This predicted interaction was validated through co-immunoprecipitation experiments (Fig. 4b), and the directly physical interaction between Siglec-15 and Beclin-1 was confirmed via co-precipitation using recombinant Beclin-1 by GST-pulldown (Fig. 4b). To identify whether Siglec-15 participates in autophagy, autophagy-related proteins were detected by Western blotting. Our results verified that Siglec-15 knockdown decreased LC3-II, ATG14 and Beclin-1 expression combined with increased p62 expression in KHOS and U2OS cells (Fig. 4c). Then, the ultrastructures during autophagy were explored using TEM. Siglec-15 silencing caused few autophagic vacuoles compared with the controls in KHOS and U2OS cells (Fig. 4d). As shown in Fig. 4e, decreased punctate LC3-II fluorescence was detected in the shSiglec-15 group cells by immunofluorescence assay, indicating the reduced expression of LC3-II in autophagosomes. In brief, our results revealed that Siglec-15 knockdown induced the inhibition of autophagy in osteosarcoma cells. The Siglec-15 expression recovery increased LC3-II and beclin-1 levels, and decreased p62 expression in shSiglec-15-KHOS cells (Additional file 2: Fig. S2a), which indicated Siglec-15 promoted autophagy (Additional file 2: Fig. S2b). In addition, Siglec-15-induced autophagy in osteosarcoma cells promotes invasion and migration of osteosarcoma cells, which can be reversed by the autophagy inhibitor 3-MA (Additional file 2: Fig. S2a–c). The quantification of Western blot results was shown in Additional file 1: Fig. S1b.

### Autophagy inhibition attenuates the migration and invasion of osteosarcoma cells by inactivating the epithelial–mesenchymal transition and affecting cytoskeletal rearrangement

**Fig. 5:**
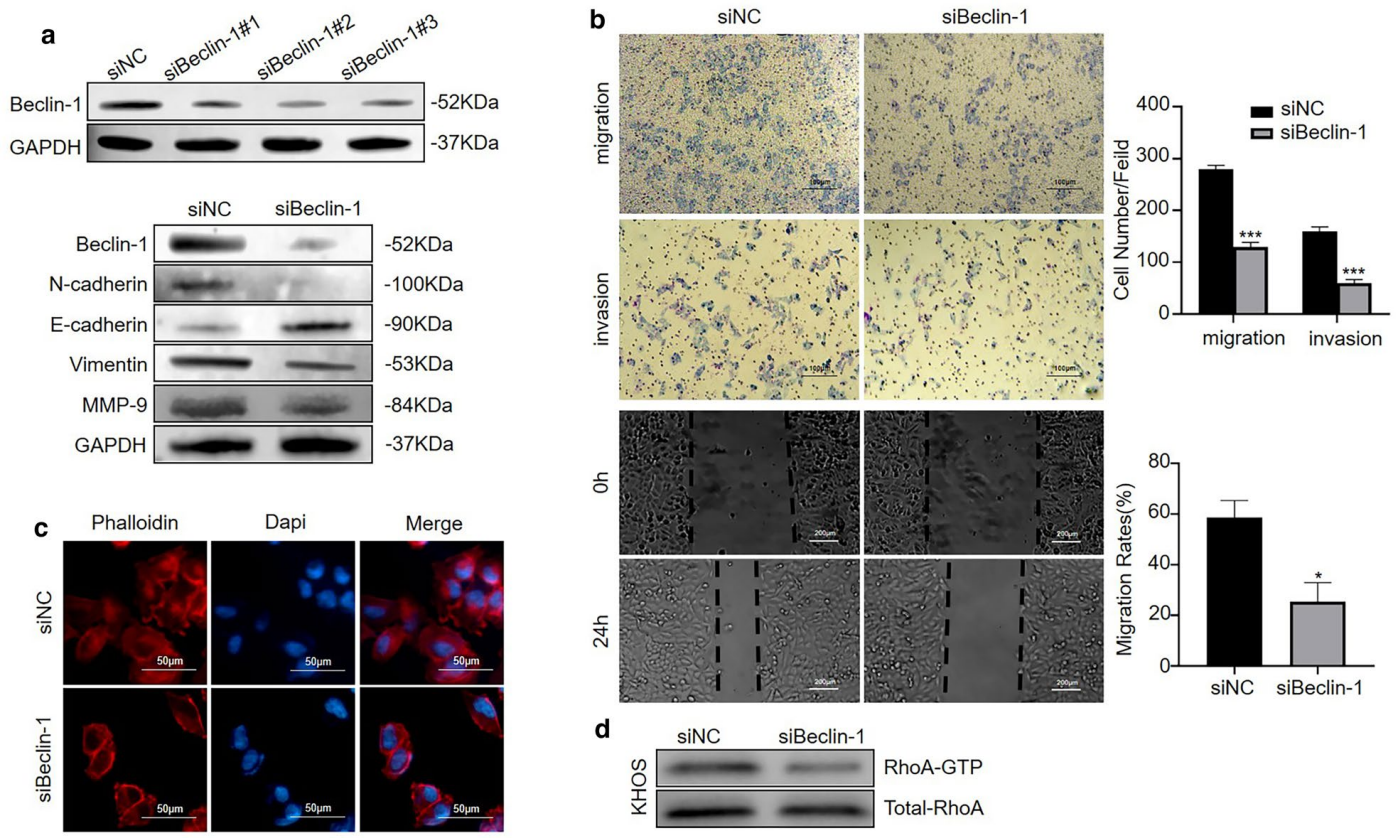
Autophagy inhibition attenuates the migration and invasion of osteosarcoma cells by inactivating epithelial–mesenchymal transition and affecting cytoskeletal rearrangement. **a** SiRNA sequences targeting Beclin-1 were used, and the knockdown efficiency was detected using Western blotting. Beclin-1 silencing inhibits the epithelial–mesenchymal transition and MMP-9 expression via Western blot. **b** Inhibition of autophagy by Beclin-1 knockdown significantly restrained the migration and invasion of osteosarcoma cells. **c** In contrast to the Beclin-1-depletion group, the lamellipodial prominences and F-actin filaments gathered near the edge of the control group. Representative images are shown. Cell nuclei are stained with DAPI. The scale bar represents 50 μm. **d** Beclin-1 silencing results in decreased RhoA activation

As a double-edged sword, autophagy can boost tumor cell metastasis and inhibit tumor metastasis [21]. To confirm the role of Siglec-15-induced autophagy in osteosarcoma cell metastasis, we knocked down Beclin-1, a crucial element for the autophagy process, in KHOS cells by siRNA. While Beclin-1 has been reported to regulate the EMT process [22], decreased expression of N-cadherin, vimentin and MMP-9 and increased expression of E-cadherin were observed after Beclin-1 silencing (Fig. 5a). Transwell and wound healing experiments proved that Beclin-1 silencing significantly weakened the migratory and invasive capacities of KHOS cells (Fig. 5b). In contrast to those in the Beclin-1-depletion group, the lamellipodial prominences and F-actin filaments gathered near the edge of the control group (Fig. 5c). Furthermore, the relationship between autophagy and RhoA, an important regulator of actin reorganization, was investigated. Beclin-1 silencing resulted in decreased RhoA activation (Fig. 5d). The quantification of Western blot results was shown in Additional file 1: Fig. S1c.

### The decreased invasion and migration caused by Siglec-15 silencing could be reversed by Beclin-1 overexpression by targeting the EMT and Beclin-1/ATG14 pathway

**Fig. 6:**
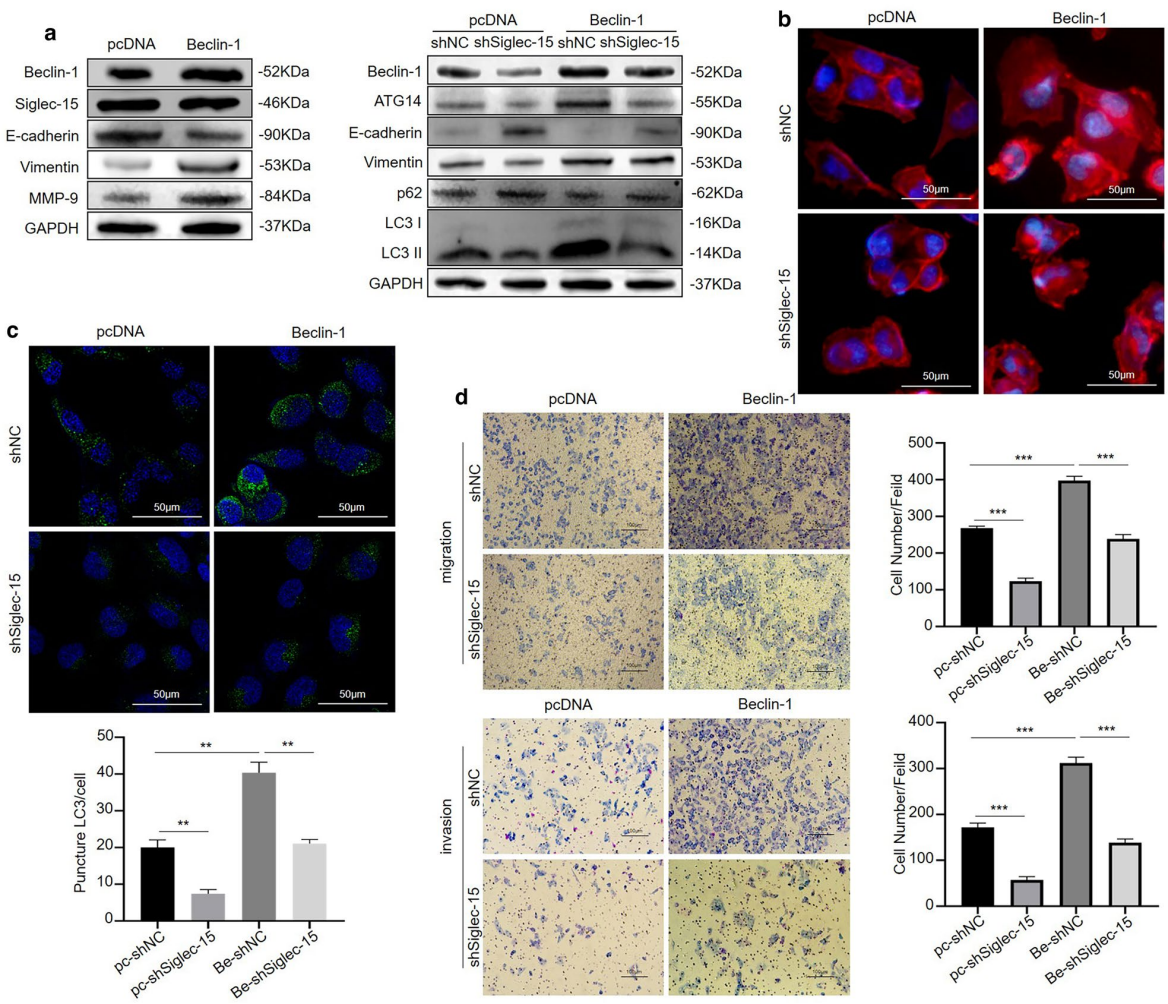
The decreased invasion and migration caused by Siglec-15 silencing could be reversed by Beclin-1 overexpression. **a** Beclin-1 overexpression increased MMP-9 levels and induced EMT, while Siglec-15 expression did not change (left). Overexpression of Beclin-1 promoted the decreased EMT and autophagy processes caused by Siglec-15 silencing, and the expression levels of related protein markers were tested by Western blotting (right). **b** Immunofluorescence assays of the cytoskeleton were used to detect changes in the cytoskeleton after Beclin-1 overexpression combined with Siglec-15 silencing. **c** An immunofluorescence assay of LC3 was used to verify changes in autophagy after Beclin-1 overexpression combined with Siglec-15 silencing. **d** Transwell assays indicate that the decreased invasion and migration caused by Siglec-15 silencing could be reversed by Beclin-1 overexpression. Data are presented as the mean ± SD from three experiments. **P < 0.01, ***P < 0.001

To further confirm the role of Siglec-15-induced autophagy in osteosarcoma cell metastasis, KHOS cells were transfected with a Beclin-1 overexpression vector, and Beclin-1 overexpression increased MMP-9 expression levels and promoted EMT, as shown by Western blotting (Fig. 6a). Additionally, the expression of Siglec-15 did not change after Beclin-1 overexpression in KHOS cells (Fig. 6a), which means that Beclin-1 was a downstream molecule of Siglec-15. Siglec-15 knockout obviously inhibited Beclin-1 overexpression-induced autophagic flux, as shown by Western blotting (Fig. 6a), and similar results were observed by LC3-II immunofluorescence (Fig. 6c). Mechanistically, Siglec-15 silencing decreased the EMT process and affected cytoskeletal rearrangement, while all these phenomena were reversed by Beclin-1 overexpression (Fig. 6a, b). The results of Transwell experiments were also consistent with the above results (Fig. 6d). Ultimately, all these findings, through both genetic inhibition and overexpression of autophagy, indicated that Siglec-15-induced autophagy promoted the invasive and migratory ability of human osteosarcoma cells by targeting the EMT and Beclin-1/ATG14 pathway. The quantification of Western blot results was shown in Additional file 1: Fig. S1d.

### Siglec-15 knockdown attenuates pulmonary metastasis of osteosarcoma cells in vivo

**Fig. 7:**
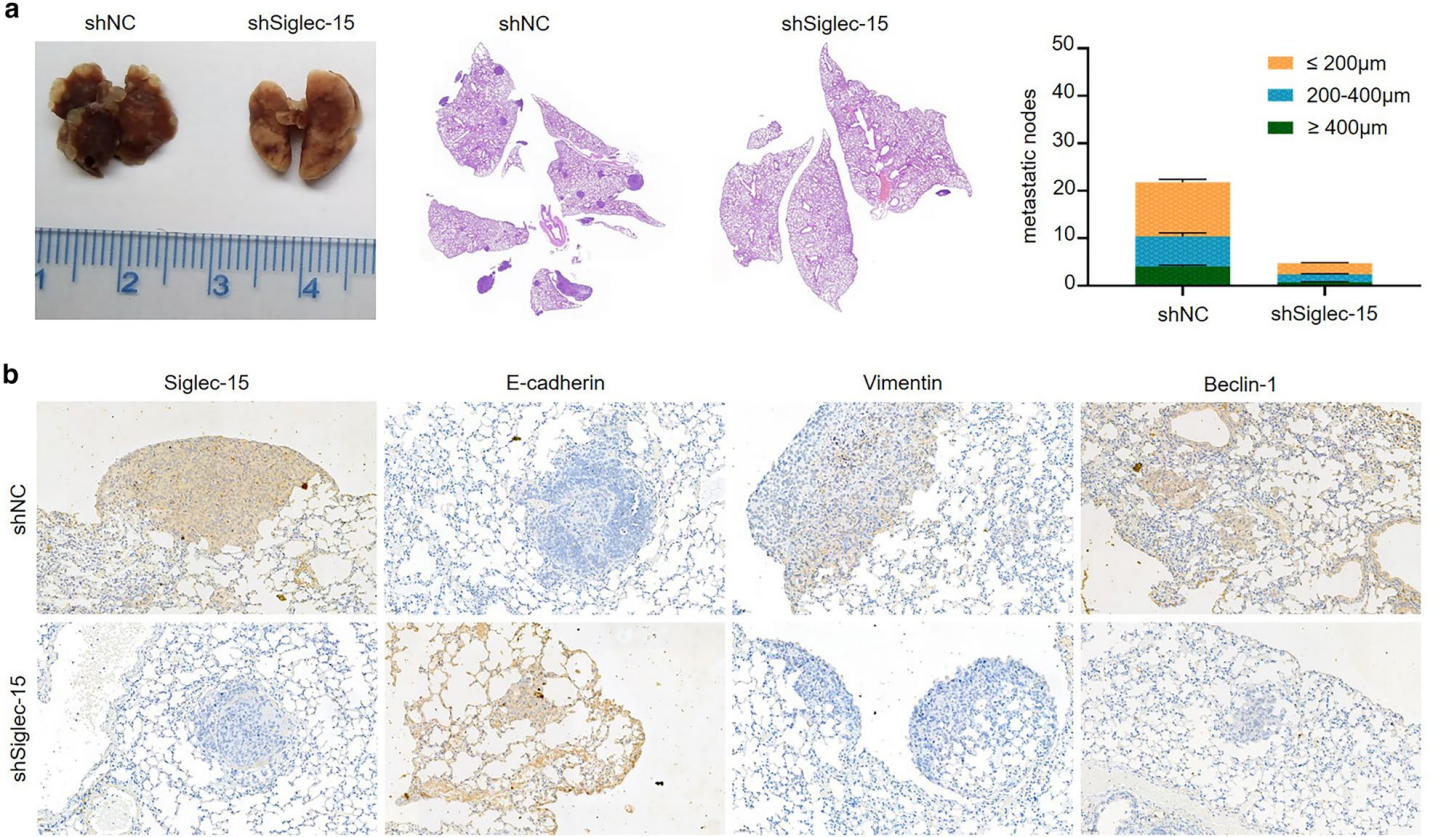
Siglec-15 knockdown attenuates pulmonary metastasis of osteosarcoma cells in vivo. **a** Representative images of lungs from the shSiglec-15 group and the control group. Different sizes of pulmonary metastases were quantitated on the basis of H&E staining results. **b** Immunohistochemistry results indicated the differences in the expression of Siglec-15, Beclin-1 and EMT markers (E-cadherin, vimentin) in lung metastases from the shSiglec-15 group and the control group. Representative images are shown at ×200 magnification

To further confirm these findings obtained in vitro, we used an in vivo xenograft model built according to the description in “Materials and methods” section. The shSiglec-15 group exhibited significantly fewer lung metastases and smaller volumes than the shNC group (Fig. 7a). Furthermore, IHC analyses of lung metastases showed elevated E-cadherin in lung metastases of the shSiglec-15 group compared with controls. Moreover, the expression of Siglec-15, vimentin and Beclin-1 was significantly decreased in lung metastases of the shSiglec-15 group compared with the control group (Fig. 7b). Taken together, these results suggested that Siglec-15 loss inhibited EMT and autophagy, through which Siglec-15 reduced osteosarcoma cell metastasis.

## Discussion

Siglec-15 was originally categorized as one of the Siglec family members. Previous studies have focused on its role in bone differentiation, bone remodeling and microbial infection [7, 23, 24]. Recently, due to the therapeutic effect of immunotherapy in numerous solid tumors, an increasing number of scholars have begun to pay attention to its immune function. Studies have confirmed that the expression of Siglec-15 is upregulated on tumor-associated macrophages (TAMs) and on tumor cells but not in normal tissues [25, 26]. Interestingly, Siglec-15 shows a similar domain composition and high homology with PD-L1, while the expression of Siglec-15 and PD-L1 is mutually exclusive in human lung cancer tissues [10]. Therefore, the Siglec-15 molecule is characterized as a potential immune suppressive molecule and may be used for immunotherapy, especially for tumor patients with low expression of PD-L1.

In recent years, some studies have found that Siglec-15 is involved in the progression of various tumors [10, 27, 28]. At present, there are few studies on this aspect of osteosarcoma, and a previous study showed that Siglec-15 is involved in the invasion and migration of osteosarcoma [12]. Our previous study examined the relationship between Siglec-15 and the apoptosis and pyroptosis of osteosarcoma cells. In this study, we first proved that Siglec-15 interacted immediately with autophagy-related proteins and was involved in the regulation of autophagy. Additionally, we demonstrated that Siglec-15 silencing reduced the expression of Beclin-1/ATG14, and Siglec-15-related autophagy could promote EMT and affect cytoskeletal rearrangement, through which Siglec-15-induced autophagy participated in the regulation of osteosarcoma cell metastasis.

Osteosarcoma is the most common primary malignant bone tumor in children and adolescents, with a high risk of pulmonary metastasis [29]. Pulmonary metastasis is a critical factor associated with poor prognosis in osteosarcoma patients. How to prevent and control pulmonary metastasis of osteosarcoma has always been an intractable clinical problem. Most studies on immunosuppressive molecules, such as Siglec-15, PD-L1, and PD-L2, have concentrated mainly on immune cells or the interaction between immune cells and tumor cells in the tumor microenvironment (TME) [30–34]. However, few studies have focused on the tumor cell intrinsic functions of these immunosuppressive molecules, especially Siglec-15, which are highly expressed in many tumor cells.

A previous study suggested that Siglec-15 expression was positively associated with lung metastasis, and dual-specificity phosphatase 1 (DUSP1) expression was positively associated with the Enneking stage [12]. Their study demonstrated that Siglec-15 promoted the migration and invasion of osteosarcoma cells via the DUSP1-inactivated MAPK pathway [12]. In our study, we demonstrated that both Siglec-15 and Beclin-1 expression was evaluated in lung metastases compared with paired primary osteosarcoma specimens using IHC. Similarly, both Siglec-15 and Beclin-1 were also highly expressed in the group with pulmonary metastasis of osteosarcoma compared to the group without lung metastasis. In addition, Siglec-15 expression was positively correlated with the expression of Beclin-1 in lung metastases. Based on these results, we hypothesized that Siglec-15 was involved in autophagy in osteosarcoma and that autophagy was closely related to pulmonary metastasis. Therefore, in vitro experiments were conducted to verify the effect of Siglec-15 on metastasis and autophagy in osteosarcoma. On the one hand, Siglec-15 depletion significantly decreased the migration and invasive capacity of osteosarcoma cells. On the other hand, the outcomes confirmed for the first time that Siglec-15 knockout inhibited autophagy in osteosarcoma cells. Next, we further investigated the possible effects of Siglec-15-induced autophagy on metastasis and the underlying mechanisms.

To explore the potential mechanisms of the Siglec-15 associations with lung metastasis, mRNA expression after Siglec-15 silencing in osteosarcoma cells was clustered and visualized using bioinformatics analysis. The differentially expressed gene patterns, gene set enrichment analysis (GSEA) and KEGG pathway enrichment analysis of Siglec-15-related genes were also investigated. The results revealed that Siglec-15 may be related to focal adhesion, adherens junction pathways. Next, we verified these results by in vitro and in vivo experiments.

The relationship between autophagy and tumor metastasis remains complex and unclear, and autophagy may be able to both restrain and accelerate tumor metastasis in different circumstances. How autophagy influences metastasis remains unclear. Both knockout and overexpression of Beclin-1 were used for osteosarcoma cells to verify the role of Siglec-15-induced autophagy in metastasis. Our studies confirmed that Siglec15-induced autophagy could promote the invasion and migration of osteosarcoma cells. Furthermore, bioinformatics analysis and coimmunoprecipitation demonstrated that Beclin-1, a pivotal autophagy protein, interacted directly with Siglec-15, consistent with the IHC results. In summary, these results suggested that autophagy was a bridge between Siglec-15 and metastasis and that Beclin-1 was an important junction.

To confirm the results of bioinformatics analysis, we examined the effect of Siglec-15-induced autophagy on cytoskeletal structure and EMT in osteosarcoma. Changes in the actin cytoskeleton have been shown to play a considerable role in cell migration and motility [20]. Actin reorganization is regulated by cofilin and LIMK, which are downstream molecules of RhoA, and their phosphorylation is regulated by RhoA activation [35]. The regulation between autophagy and the RHO family has double-planedness in some studies [36, 37]. Herein, we found that both Siglec-15 silencing and Beclin-1 depletion could decrease RhoA activation and lessen the formation of lamellipodial protrusions in the submembranous area in osteosarcoma cells. Moreover, the increased lamellipodial protrusions induced by Beclin-1 overexpression can be rescued by Siglec-15 knockdown. Thus, Siglec15-induced autophagy is a prometastatic mechanism that affects cytoskeletal rearrangement.

Previous studies revealed the influence of autophagy on EMT regulation [38, 39]. Similar to its double-sided effect on the tumor, the effect of autophagy on the EMT process was also complicated depending on the cellular condition [40]. Our data clearly showed that Beclin-1 depletion resulted in reduced migration of osteosarcoma cells by suppressing EMT, and the increased migration caused by Beclin-1 overexpression could be reversed by Siglec-15 silencing. Therefore, the autophagy produced by Siglec-15 promoted metastasis by facilitating EMT.

Overall, to the best of our knowledge, studies on the relationship between Siglec-15 and autophagy are scarce. Our study demonstrated that Siglec-15 could interact immediately with Beclin-1 and regulate autophagy. In addition, the autophagy induced by Siglec-15 could promote EMT and affect cytoskeletal rearrangement through the effect of these two pathways, and Siglec-15-induced autophagy promoted the invasion and metastasis of human osteosarcoma cells via the Beclin-1/ATG14 pathways.

## Conclusions

In summary, our study reveals for the first time the prometastatic mechanism of Siglec15-induced autophagy in osteosarcoma. Siglec-15-induced autophagy promotes migration and invasion by targeting Beclin-1/ATG14 pathways and activating EMT both in vitro and in vivo, and Siglec-15-induced autophagy extends our comprehension of the regulation of autophagy on tumor metastasis and provides a potential target for metastatic osteosarcoma treatment.

## Supplementary Information

**Additional file 1: FigureS1.** The quantificationof Western blots results in Figs. 3a, 4c, 5a and 6a. (a) The quantification ofWestern blot results in Fig. 3a. (b) The quantification of Western blot resultsin Fig. 4c. (c) The quantification of Western blot results in Fig. 5a. (d) Thequantification of Western blot results in Fig. 6a. Data are presented as themean ± S.D. (**P < 0.01).

**Additional file 2: FigureS2.** Effect of Siglec-15 expressionrecovery on migration, invasion and autophagy of osteosarcoma cells. (a) Westernblots were used to detect the expression of EMT and autophagy-related proteinsin shSiglec-15-KHOS cells recovering Siglec-15 with or without 3-MA treatment. (b) Cells after Siglec-15 expression recovery increased apunctate pattern of LC3-II fluorescence. shSiglec-15-KHOS cells were incubatedwith or without 3-MA. (c) Transwell assay was used to detect the invasion andmigration ability of shSiglec-15-KHOS cells with or without 3-MA treatment onthe basis of Siglec-15 expression recovery. These experiments were repeated 3times. Data are presented as the mean ± S.D. (**P < 0.01).

## Data Availability

The RNA-sequencing data presented in this study can be found in the Sequence Read Archive (SRA) (accession no. PRJNA832814).

## Abbreviations

Siglec-15: Sialic acid-bound immunoglobulin lectin 15
PD-L1: Programmed cell death ligand 1
PD-L2: Programmed cell death ligand 2
PD-1: Programmed cell death 1
TIM-3: T-cell immunoglobulin and mucin domain-3
LAG-3: Lymphocyte-activation gene 3
TAMs: Tumor-associated macrophages
TME: Tumor microenvironment
DUSP1: Dual-specificity phosphatase 1
IHC: Immunohistochemistry
EMT: Epithelial–mesenchymal transition
ATG14: Autophagy-related genes 14
KEGG: Kyoto Encyclopedia of Genes and Genomes
GSEA: Gene set enrichment analysis
