# Supplementary material for: Siglec-15-induced autophagy promotes invasion and metastasis of human osteosarcoma cells by activating the epithelial–mesenchymal transition and Beclin-1/ATG14 pathway
Source: Cell Biosci. 2022 Jul 16;12:109. doi: 10.1186/s13578-022-00846-y (PMC9287887; doi:10.1186/s13578-022-00846-y)
Supplement: Supplementary file 1 — Additional file 1: FigureS1. The quantificationof Western blots results in Figs. 3a, 4c, 5a and 6a. (a) The quantification ofWestern blot results in Fig. 3a. (b) The quantification of Western blot resultsin Fig. 4c. (c) The quantification of Western blot results in Fig. 5a. (d) Thequantification of Western blot results in Fig. 6a. Data are presented as themean ± S.D. (**P < 0.01). [file 13578_2022_846_MOESM1_ESM.docx]

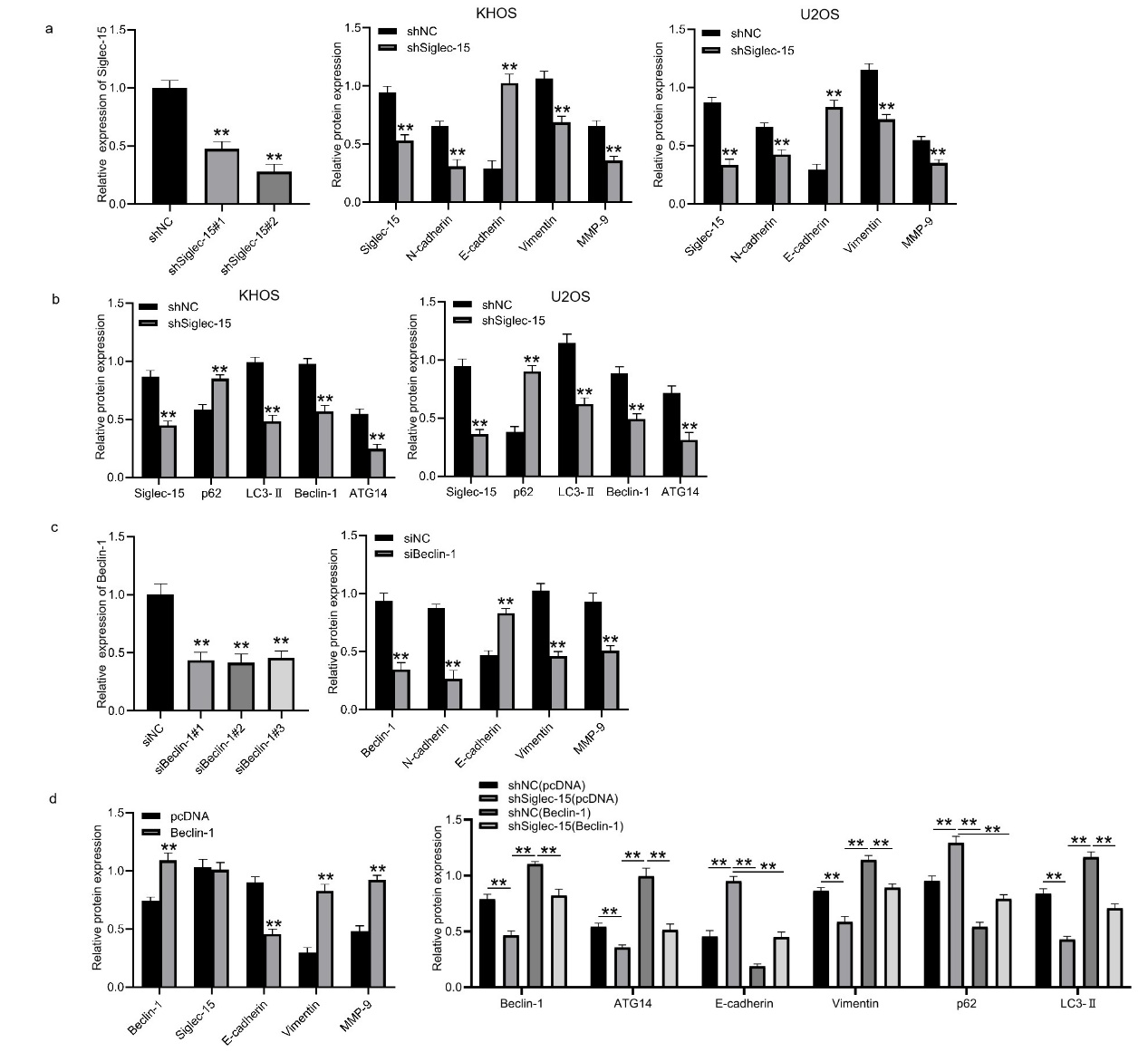


Figure S1: The quantification of Western blots results in Fig 3a, 4b, 5a and 6a. (a) The quantification of Western blot results in Fig. 3a. (b) The quantification of Western blot results in Fig. 4b. (c) The quantification of Western blot results in Fig. 5a. (d) The quantification of Western blot results in Fig. 6a. Data are presented as the mean ± S.D. (***P*＜0.01).
