# Supplementary material for: Siglec-15-induced autophagy promotes invasion and metastasis of human osteosarcoma cells by activating the epithelial–mesenchymal transition and Beclin-1/ATG14 pathway
Source: Cell Biosci. 2022 Jul 16;12:109. doi: 10.1186/s13578-022-00846-y (PMC9287887; doi:10.1186/s13578-022-00846-y)
Supplement: Supplementary file 2 — Additional file 2: FigureS2. Effect of Siglec-15 expressionrecovery on migration, invasion and autophagy of osteosarcoma cells. (a) Westernblots were used to detect the expression of EMT and autophagy-related proteinsin shSiglec-15-KHOS cells recovering Siglec-15 with or without 3-MA treatment. (b) Cells after Siglec-15 expression recovery increased apunctate pattern of LC3-II fluorescence. shSiglec-15-KHOS cells were incubatedwith or without 3-MA. (c) Transwell assay was used to detect the invasion andmigration ability of shSiglec-15-KHOS cells with or without 3-MA treatment onthe basis of Siglec-15 expression recovery. These experiments were repeated 3times. Data are presented as the mean ± S.D. (**P < 0.01). [file 13578_2022_846_MOESM2_ESM.docx]

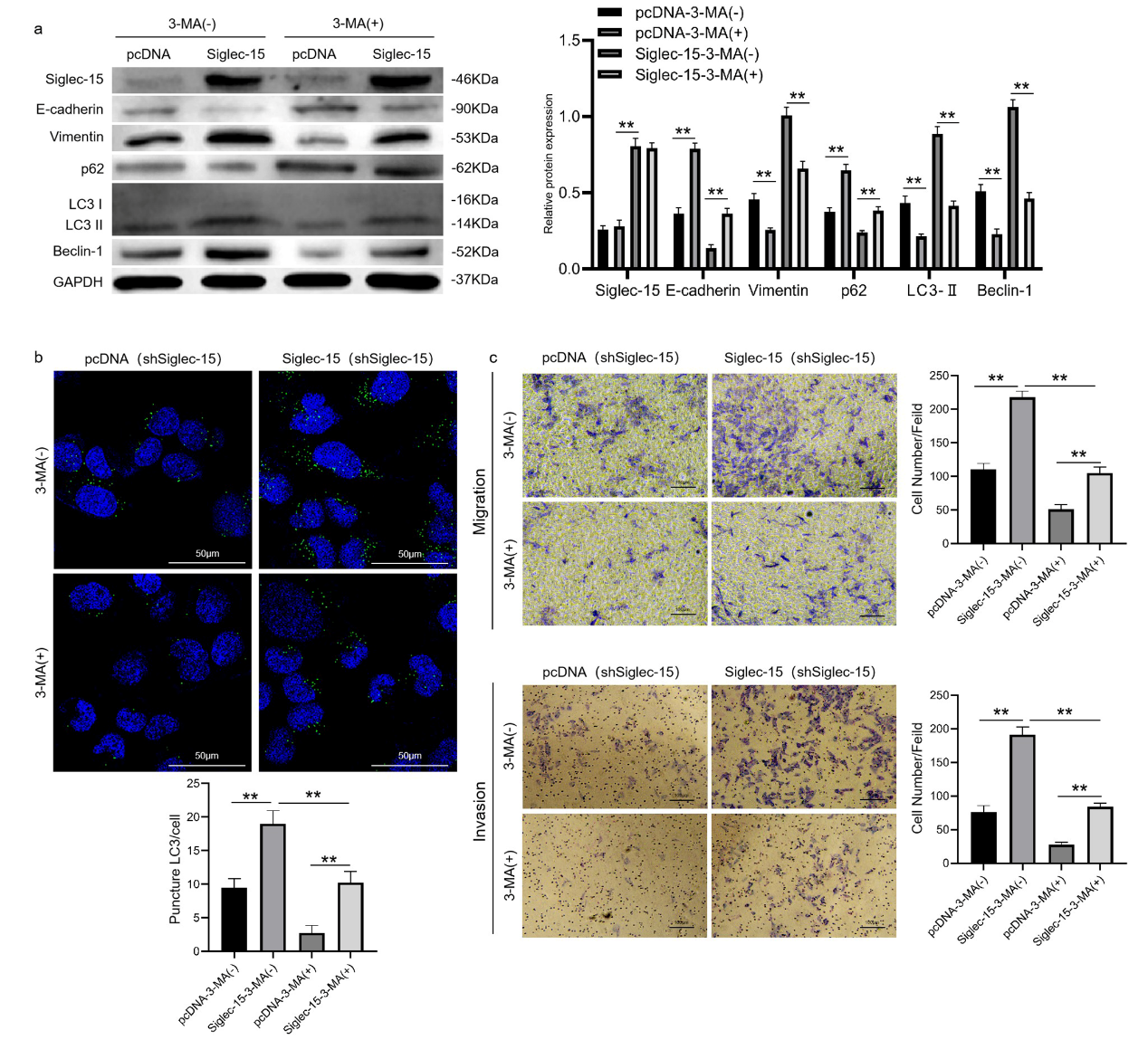


Figure S2: Effect of Siglec-15 expression recovery on migration, invasion and autophagy of osteosarcoma cells. (a) Western blots were used to detect the expression of EMT and autophagy-related proteins in shSiglec-15-KHOS cells recovering Siglec-15 with or without 3-MA treatment. (b) Cells after Siglec-15 expression recovery increased a punctate pattern of LC3-II fluorescence. shSiglec-15-KHOS cells were incubated with or without 3-MA. (c) Transwell assay was used to detect the invasion and migration ability of shSiglec-15-KHOS cells with or without 3-MA treatment on the basis of Siglec-15 expression recovery. These experiments were repeated 3 times. Data are presented as the mean ± S.D. (***P*＜0.01)
